# Significance of Whole Blood Viscosity in Acute Ischemic Stroke

**DOI:** 10.3390/life15121869

**Published:** 2025-12-05

**Authors:** Irena Velcheva, Nadia Antonova, Tsocho Kmetski

**Affiliations:** 1 Department of Neurology, University Hospital Acibadem City Clinic, Okolovrasten Pat Str. 127, 1407 Sofia, Bulgaria; ts.kmetski@gmail.com; 2 Department of Biomechanics, Institute of Mechanics, Bulgarian Academy of Sciences, Akad. G. Bonchev Str. Bl. 4, 1113 Sofia, Bulgaria

**Keywords:** acute ischemic stroke (AIS), whole blood viscosity (WBV), WBV measurements

## Abstract

The paper provides a comprehensive review of the relationship between whole blood viscosity (WBV) and acute ischemic stroke (AIS) concerning AIS risk and type, and its treatment and prognosis. A significant increase in diastolic blood viscosity (DBV) at the onset of AIS was established in the small-artery occlusion stroke subtype. In patients with atherothrombotic causes of AIS, systolic (SBV) and DBV values were higher than in those with an embolic cause. The higher WBV at low shear rates on hospital admission is associated with an increased risk of early neurological deterioration and disease progression in the patients with AIS. Most studies reveal the association of increased WBV at the stroke onset with poor functional outcome after applying intravenous thrombolysis or endovascular thrombectomy. However, significant reduction in WBV after the combined use of these therapeutic methods in AIS patients was observed. Whole blood viscosity has an obvious effect on the risk of AIS, its clinical severity and outcome. Further research is needed due to the multiple devices and techniques used, like cone–plate viscometers, scanning capillary viscometers, EMS viscometers, parallel-plate rheometers and the different associations of WBV with some of the applied treatment strategies.

## 1. Introduction

Stroke is the second leading cause of death globally [1] and ischemic stroke represents 85% of all stroke cases [2]. In 2021, the global burden of ischemic stroke is presented with a 69.9 million prevalence and 7.8 million incident ischemic strokes [1]. Among the pathophysiological factors for cerebral ischemia, whole blood viscosity (WBV) has a certain significance for the development of ischemic stroke. This is accomplished through its influence on cerebral blood flow and cerebral perfusion [3] and its role in atherosclerosis and thrombogenesis.

The study aims to provide a comprehensive overview of the changes in blood viscosity in acute ischemic stroke (AIS), as well as to analyze these changes depending on the methods and measuring devices used. The analysis of the effects of WBV is important to assess its influence on the occurrence of stroke and on the disease prognosis.

## 2. Whole Blood Viscosity in the Pathophysiology of AIS

Whole blood viscosity is involved in the pathophysiology of AIS because of its adverse effects on blood flow and on perfusion through the brain-supplying arteries, resulting from thrombotic and embolic conditions.

Whole blood viscosity is the resistance to flow in blood vessels, developed by the internal frictional forces between adjacent layers of fluid in relative motion. Its major determinants are hematocrit (Hct), plasma viscosity (PV), erythrocyte aggregation (EA) and erythrocyte deformability (ED). Blood behaves as a non-Newtonian fluid in the circulation, and it changes in a non-linear relationship depending on the shear rate [4]. In the blood vessels, WBV is an important component of vascular wall shear stress (WSS) and contributes to the occurrence and development of atherosclerotic lesions [5,6]. The WSS, the friction force exerted parallel to the vessel wall, is directly proportional to WBV and is one of the primary determinants of the endothelial cell function [7,8]. The interaction of hyperviscous blood may cause structural and biochemical alterations in the capillary endothelium [9], leading to endothelial remodeling and altered vascular physiological responses [5]. Endothelial cells respond to shear force changes by altering their morphology, proliferation and gene expression, promoting lipid accumulation and oxidation, inflammatory cell infiltration, smooth muscle cell proliferation and extracellular matrix production. The low WSS, a factor for plaque formation, induces high expression of adhesion molecules like ICAM-1, VCAM-1, MCP-1, E-selectin and cytokines and local endothelial swelling [7,8,9]. The changes in wall shear stress regulate the nitric oxide-dependent flow-mediated vasodilatation and thus influence the tissue perfusion [10]. Whole blood viscosity also influences the basal cerebral perfusion, and this was reported by Gyawali et al. [3], who found a significant correlation of the WBV at 20 s^−1^ with the CT perfusion parameters and the diffusion-weighted magnetic resonance imaging (MRT DWI) volume.

The involvement of WBV in thrombosis and hemostasis occurs mainly through its determinants: erythrocytes and fibrinogen (Fib), and their interaction. The hemorheological effects related to thrombosis include a rise in Hct, an increase in EA, mediated mainly by Fib and immunoglobulins, and a decrease in ED with alteration of the resistance to flow [11]. In the microcirculation, fibrinogen stabilizes erythrocyte aggregation and leads to an increase in WBV [12]. Erythrocytes also have effects on platelet reactivity; they interact with the vessel wall and take part in the structure and properties of clots [11]. The mechanism by which erythrocytes influence platelet activity involves rheological effects, cellular interactions, biochemical signaling and clot structuring: RBCs influence blood viscosity and how platelets move to the vessel walls (platelet margination). RBCs interact with platelets and endothelial cells, affecting their function and releasing molecules like adenosine diphosphate (ADP) that activate platelets, and their membranes contain components like phosphatidylserine that are important in the coagulation cascade. Finally, RBCs are integral to the structure of a blood clot, and their compression contributes to the formation of a tight seal. So, in patients with AIS, parallel alterations of the hemorheological and coagulatory parameters are observed [13].

## 3. Whole Blood Viscosity and the Risk of Stroke

A significant association of blood viscosity and hematocrit with stroke occurrence was established in a prospective 5-year follow-up study of a population sample by G.D.O. Lowe et al. [14]. The authors also found a significant association between PVand Fib for total cardiovascular events and stroke. In another study of 378,210 individuals, a Mendelian randomization study did not reveal a causal effect of WBV on ischemic stroke [15].

The risk factors for stroke are closely related to WBV. In a population-based cohort, a linear positive association was established between WBV and blood pressure [16]. In patients with heart failure, the higher value of WBV is associated with a higher occurrence of cardiovascular diseases (CVDs) and lower survival rates [17], so in these patients, WBV was found to be a predictor of long-term IS [18]. Also, in patients with non-valvular atrial fibrillation, increased WBV at high and low shear rates shows significant correlations with elevated CHA2DS-VA and CHA2DS2-VASc scores as indicators of higher thromboembolic risk [19]. Another risk factor for AIS is the co-occurrence of diabetes mellitus, and it is characterized by increased WBV and reduced blood flow, especially in the microcirculation. The increase in WBV is induced by the elevated blood glucose oxidative stress, leading to the alteration of erythrocyte morphology and function [20]. The increased WBV and plasma viscosity in patients with transient ischemic attacks correlate with increased blood pressure (bp) values, age, leucocyte count, fibrinogen and cholesterol [21]. When discussing WBV and stroke risk, we should note the observations of Tsuda et al. [22] and Li et al. [23], who reported an increase in WBV at low shear rates in subjects with silent cerebral infarctions with ischemic lesions on brain imaging but without clinical symptoms. The authors point out that the early measurement of WBV may help to assess the risk of stroke.

## 4. Whole Blood Viscosity in Acute Ischemic Stroke

### 4.1. WBV at the Onset of AIS and During Its Follow-Up

Most investigations concerned the significance of WBV and other hemorheological parameters in acute ischemic stroke [3,22,24,25,26,27,28,29,30,31,32,33,34,35,36,37,38,39,40] (Table 1).

The measurement of WBV was usually performed within 12 to 72 h after the stroke symptom onset and the following examinations were within 5 days to 3 months after the acute stroke treatment. Some of the studies included patients with transient ischemic attacks (TIAs) [25,41,42]. In addition to WBV, other hemorheological variables like PV, EA and ED were examined [24,25,38] and their association with the Fib values was estimated [24,25,26,30,41].

In a great number of the acute stroke cases, the WBV values during the onset of stroke were significantly increased in comparison to the control groups [22,24,25,26,27,28,29,30,34], except for the studies of Uygun et al. [38] and Noh et al. [39]. In all studies where EA and ED were estimated, a significant increase in EA was detected [25,26,28,38]. Regardless of the time of the follow-up measurement, which was on the fifth day [38], the first week [30], the third week [26], the first month [22], second month [25] or third month [5,26], a trend of normalization or significant decrease in the initial BV values was observed. Only the study of Wong et al. [26] revealed a persisting increase in WBV after three months. Song et al. [30], who found a subsequent increase in WBV on the fifth week after the initial decrease, supposed that the decrease in WBV during the first week might be related to applied hydration therapy. Some authors pay special attention to the existing dehydration in part of the patients at the onset of AIS and its association with increased WBV [29,30,40] and they propose the investigation of the blood urea nitrogen/creatinine ratio as a marker of the dehydration state.

Most of the authors described the examined hematological and biochemical variables in patients with AIS. However, few of them were mentioned to correlate with WBV: Fib values and albumin/globulin ratio [24], WBC count, platelet count and HbA1c [29], C-reactive protein [39].

### 4.2. Association of WBV with Stroke Risk, Etiology and Imaging

The frequency of the risk factors was presented in the clinical studies of patients with AIS. However, only Noh SM [39] reported a significant association of diastolic and systolic blood viscosity with hypertension and age.

An attempt was made to compare WBV values in the etiological subgroups of acute ischemic stroke according to the TOAST classification [43]. When estimating the hemorheological variables in the different etiologic subtypes of stroke predominance, an increase in WBV in the group with small-vessel occlusion (SVO) was reported [22,25,29,30,39,40], while other authors found it higher in strokes of cardioembolic (CE) and cryptogenic or undetermined (UND) etiology [6,25]. Additionally, increased EA in the large-artery atherosclerosis (LAA) subgroups [25,38] was found. Oh et al. [35] divided patients with strokes of UND etiology into groups with potential atherothrombosis, where systolic (SBV) and diastolic blood viscosity (DBV) values were higher than those in the group with possible embolism. The association of increased viscosity with imaging-derived stroke mechanisms in middle cerebral artery infarctions was revealed by Woo H. et al. [36] and it concerned the in situ thrombo-occlusion. As for the relationship of WBV with imaging biomarkers, DBV was found to be significantly higher in patients with old LI and microbleeds on MRI or CT [39] and to correlate with the number of chronic lacunes [30]. Also, a significant correlation of WBV with the extent of the AIS [31], the CT perfusion parameters and MRI DWI was established [3].

### 4.3. WBV and AIS Prognosis

When estimating the prognostic value of WBV, it was found that low shear viscosity was related to early neurological deterioration in patients with in situ thrombo-occlusion and in those with artery-to-artery embolism and local branch occlusion of the middle cerebral artery [36]. Lee et al. [33] announced that the occurrence of early neurological deterioration was observed in 1.5% of patients admitted for lacunar infarction. In them, DBV was significantly higher compared to patients without early neurological deterioration. In the same patients’ group, SBV and DBV were associated with the worsening of NIHSS score and progression of stroke [32]. Diastolic blood viscosity was also found to predict poor 3-month functional outcome in AIS patients [37]. Hashem et al. [31] failed to find a relationship between BV and stroke outcome; however, they found significant correlation between WBV and the extent of the cerebral infarction.

## 5. WBV Measurement in AIS

In recent years, the changes in WBV in acute ischemic stroke were estimated with different viscometers, and in some of the studies, WBV was calculated by the De Simone formula [44], using the values of Hct and total protein. Earlier measurements were performed with rotational cone–plate viscometers [24,25,26] and recent studies by scanning capillary–tube viscometers [30,33,35,36,37,39] and parallel-plate rheometers [34]. The reason is the need for point-of-care measurement after the patients’ admission with small whole blood sample and rapid performance. The investigation in patients with AIS could help to assess the severity of the disease, predict the stroke outcome, to make decisions for the treatment and estimate its effectiveness.

Formula-based viscosity (De Simone) offers quick, cost-effective estimation, but it assumes uniformity and does not reflect the dynamic shear-thinning behavior of real blood (Table 2). Viscometer-based methods (rotational, capillary, EMS, oscillatory) provide actual viscosity values across shear rates, which is critical for clinical accuracy, especially for stroke management. No study in this text directly compared calculated vs. measured viscosity side-by-side in the same patient cohort, but such comparisons would be clinically valuable for validating formulas.

## 6. Whole Blood Viscosity and AIS Treatment

Since several studies confirm the adverse effect of WBV in patients with cerebral ischemic stroke, a question arises about its relationship with the ischemic stroke treatment modalities. Bearing in mind the contemporary guidelines for the treatment of AIS [45], the changes in WBV during different treatment strategies have been investigated.

### 6.1. WBV and Thrombolytic Therapy

The association between WBV and its determinants with thrombolytic therapy was investigated in the experimental study by M. Hitosugi et al. [46], who measured it in blood samples before and after mixing with recombinant tissue plasminogen activator (alteplase) at different concentrations. The authors established that WBV’s initial increase was followed by a decrease and stabilized at a level below the initial values. A study by Rasyd AL. et al. [47] found an increase in WBV in 88.6% of the examined patients with AIS on admission. These patients had poorer neurological deficits on day 7 after intravenous thrombolysis and poor outcomes on day 30. This poor outcome was more pronounced in patients aged >65 years old, of dehydrated state and partial anterior circulation infarct. When estimating the markers of dehydration status, Li S. et al. [48] found that it is an independent risk factor for the long-term prognosis of thrombolyzed patients with acute ischemic stroke. The increased fibrinogen, a determinant of WBV, was also shown to be associated with poor response in AIS at 24 h [49] and 14 days [50] after intravenous thrombolysis. After adjustment for other stroke risk factors, increased fibrinogen was identified as an independent factor for a poor response to thrombolysis [49]. The initial neutrophil-to-lymphocyte ratio also influenced this poor response.

### 6.2. WBV and AIS Endovascular Therapy

The outcome of another contemporary treatment option for AIS—endovascular thrombectomy is also associated with the initial WBV values in AIS patients with large cerebral artery occlusion. The elevated WBV at both high and low shear rates, calculated using De Simone’s formula, proved to be an independent predictor for poor clinical outcomes on day 90 after thrombectomy [51]. In AIS patients with large-artery occlusion, high values of initial DBV were associated with the failure of first-pass reperfusion and the need for more passages during thrombectomy [52]. Another recent study on AIS patients did not reveal correlation between WBV at high and low shear rates and the functional outcome of mechanical thrombectomy [53]. Similarly to WBV, increased Fib on admission is associated with poor 3-month outcomes after endovascular thrombectomy [54]. When following the examination of WBV, PV, Fib and Hct before and 3 months after combined treatment of AIS with intravenous thrombolysis and endovascular thrombectomy, Wu L. et al. [55] observed a significant reduction in their values as compared to those after intravenous thrombolysis alone. In patients with acute ischemic stroke receiving combined treatment with intravenous thrombolysis and endovascular thrombectomy, the red blood cell fraction within the retrieved thrombi was proven to affect the thrombolytic response [56].

### 6.3. WBV and AIS Drug Treatment

Among the interactions of WBV with various therapies in AIS, its relationship with antithrombotic treatment in non-valvular atrial fibrillation is of special interest. When estimating the impact of prior antithrombotic use (antiplatelets, vitamin K antagonists and new oral anticoagulants) on WBV in cardioembolic stroke with non-valvular atrial fibrillation, Jung et al. [57] indicated their significant association with decreased SBV and DBV in parallel with Hct values, similar to the results of the same scientific team in a group of AIS and TIA patients with predominating lacunar stroke [58], where antithrombotic treatment included aspirin, clopidogrel and warfarin. When comparing the effect of the different antithrombotic drugs on WBV in acute cardioembolic stroke, warfarin showed greater reduction in WBV at all shear rates than aspirin [59]. The effect of the new oral anticoagulants did not show a difference compared to warfarin [57], and this is particularly favorable because of their convenience of use and high efficacy in reducing intracranial hemorrhage [60]. As for aspirin, its association with WBV showed different responses. Lee C. et al. [59] and Rosenson R. et al. [61] did not show changes in WBV after aspirin treatment. The response to aspirin was better when it was combined with clopidogrel [62], and this combination could provoke a significant reduction in WBV in AIS [57,58]. A positive influence of clopidogrel as an inhibitor of platelet function on the hemorheological profile was indicated [62,63]. In patients with ischemic stroke or transient ischemic attacks, clopidogrel was related to a decrease in SBV, depending on their CYP2C19 genotype status [64], and lowering of BV could be due to the increase in adenosine and cyclic adenosine monophosphate plasma concentration [63].

During the years, the pharmacological agents aimed at influencing whole blood viscosity and improving cerebral blood flow are nimodipine, vinpocetine and pentoxifylline, pentoxifylline being the most extensively researched among them. Its properties to reduce Fib, PV and WBV, to diminish erythrocyte and platelet aggregation and to increase blood filterability led to improvement in blood flow in the microcirculation [65]. A meta-analysis of studies in patients with acute stroke receiving intravenous and oral pentoxifylline revealed a strong trend toward reduction in early mortality [66]. A parallel decrease in BV with improved clinical outcome was reported by Rasyd A. et al. [67], but the data again did not reach statistical significance. Another drug agent with possible hemorheological effect is vinpocetine, which has been shown to increase the elasticity of the erythrocyte membranes and their deformability in parallel with lowering the degree of disability and cognitive functions in acute stroke [68]. Despite its promising effects, there was not enough evidence to suggest it could reduce case fatality and its routing administration in patients with acute ischemic stroke was not recommended [69].

Regarding the calcium antagonist nimodipine, an experimental study revealed an improvement in brain energy metabolism and blood rheology with a significant decrease in WBV at high shear rate during ischemia and reperfusion in the gerbil brain [70]. A similar beneficial effect of nimodipine on plasma fibrinogen concentration and blood viscosity at low shear 3 weeks after acute ischemic stroke was found by Ameriso et al. [71]. In addition to its hemorrhagic effects, nimodipine has been discussed for reducing vasospasm and microthromboembolism, increasing fibrinolytic activity, and neuroprotection; however, it has not been proposed for the treatment of acute stroke due to the drug-induced significant decrease in systolic and diastolic blood pressure with subsequent poor outcome [72].

## 7. Conclusions

The review highlights important limitations in the current understanding of whole blood viscosity (WBV) in acute ischemic stroke (AIS). Studies have used a variety of measurement techniques and devices, resulting in inconsistent shear rate conditions, sample processing, and hematocrit corrections, which limit comparability. The time to WBV assessment also varies considerably—from hours after the onset of AIS to weeks or months—complicating the interpretation of time trends and the influence of hydration or treatment. Evidence regarding etiology-specific patterns remains conflicting, with some studies reporting increased WBV in small-vessel occlusion and others in cardioembolic or unspecified subtypes. Prognostic findings show similar variability, with WBV being associated with functional outcome and early neurological deterioration in some cohorts but not in others.

Future research should focus on standardizing measurement protocols, harmonizing the timing of assessments, and incorporating WBV into larger longitudinal studies. Integration with advanced neuroimaging and clearer etiologic stratification may improve mechanistic understanding. Additionally, evaluating whether targeted rheological interventions or hydration strategies can improve thrombolytic or endovascular outcomes could clarify the therapeutic relevance of WBV in AIS management.

## Figures and Tables

**Table 1:** Key observations on whole blood viscosity (WBV) changes in ischemic stroke groups.

Ischemic Stroke Groups	Hemorheological Tests	Venepuncture and Measurement Time After AIS Onset	WBV Measurement Method/Device Shear Rate	Key Observations	Authors
AIS, TIAs, stroke risk group	WBV PV	≤1 h after venepunctute with EDTA	Contraves LS viscometer; Ostwald microviscometer 0.145–124 s^−1^ at 25 °C	Increased WBV and PV Correlation with elevated FIB and albumin/globulin ratio	Coull, B. et al. [24]
AIS: large vessel, lacunar, cardiogenic	WBV, PV EA, ED	Within 72 h with EDTA and 2 mos later Up to 5 h testing	Wells-Brookfield Micro Cone-Plate Viscometer 75 s^−1^–1500 s^−1^, at 25 °C WBV at 40% Hct Zeta sedimentation ratio Centrifugal deformability technique	Increased WBV, PV and EA Increased WBV for cardiogenic and lacunar AIS with a trend for decrease after 2 months	Fisher, M. et al. [25]
AIS	WBV, PV, Ht, EA, ED	After 3 wks and 3 mos	Brookfield Cone-Plate viscometer at 35 °C	Increased BV, PV and EA, persisting on 3rd wk and 3 mo	Wong, W.J. [26]
SI; AIS-lacunar Chronic lacunar— 12.5 mos after AIS	WBV, PV	Within 3 days of onset and after 1 month	Cone-plate viscometer WBV corrected to 45% Ht, 22.5 s^−1^–225.0 s^−1^	Increased WBV and PV in acute LI; increased WBV after 1 mo and in chronic LI	Tsuda, Y. et al. [22]
AIS Chronic IS— 3–6 mos after AIS	PV, Rel. BV = WBV/PV, Shear stress	Within 12 h after onset with EDTA Up to 5 min testing	Rotational-oscilatory reometer Contraves LS40 at 37 °C 0.01 s^−1^–100 s^−1^	Increased relative BV in AIS and less pronounced in chronic IS. Increased PV in both groups.	Kowal, P. et al. [27]
AIS	WBV, PV EA, ED	4 h after venepunctue in heparinized tubes	Capillary viscometer with optoelectronical detection of flow, Microscope with digital camera, Micropore filtration system	Increased BV, PV and EA, decreased ED. BV correlated with the microcirculatory parameters of the upper forearm.	Tikhomirova, I. et al. [28]
AIS: CE, LAA, SAO	WBV	0.3 mL sample with EDTA on admission day, after 1 wk, 2 wks	Electromagnetic spinning sphere viscometer (EMS) at 37 °C, 100 s^−1^	Significantly increased BV in SAO. The increased BV in SAO, CE and LAA is reduced on the 1st wk and increased on the 2nd wk (contribution of dehydration).	Furukawa, K. et al. [29]
AIS: CE, LAA, SAO, Cryp, stroke mimic	SBV DBV	3 mL sample with EDTA within 3 days of onset after 1 wk, 5 wks Up to 24 h testing	Scanning capillary tube viscometer (BVD-PRO1) 1 s^−1^–300 s^−1^	DBV highest in SAO. It decreased on the 1 wk and increased on the 5 wk (contribution of dehydration). DBV correlated with the number of chronic lacunes on MRI.	Song, S.H. et al. [30]
AIS	BV Hct	Within 24 h from onset before IV infusion	Ostwald glass capillary viscometer	BV was not a significant predictor of AIS outcomes BV correlated with the size of cerebral infarction on MRI	Hashem, S. et al. [31]
LI	SBV DBV	Within 5 days of AIS onset before IV infusion Up to 24 h testing	Scanning capillary viscometer (Hemovister) 1 s^−1^–300 s^−1^	Higher DBV at admission is associated with increased risk of progressive stroke in men.	Han, S. et al. [32]
AIS: CE, LAA, SVO, UD	WBV	5 mL EDTA sample before treatment Up to 2 h testing	Brookfield DVII viscometer with CP40 spindle at 37 °C; WBV adjusted to 40% Ht; 20 s^−1^	Higher WBV in CE and UD AIS. Correlation of WBV with CT perfusion parameters and MRI DWI volume.	Guawali, P. et al. [3]
LI	SBV DBV	Sample with EDTA within 24 h	Scanning capillary viscometer (Hemovister) 5 s^−1^–300 s^−1^	Increased DBV is associated with early neurological deterioration of LI in the anterior circulation.	Lee, H. et al. [33]
AIS before and after IV fluid, hemorrhagic stroke, stroke mimics	WBV	2 mL sample without anticoagulants Up to 3 min testing	Parallel plate rheometer 1, 5, 10 rad/s (oscillation)	Increased WBV when compared to stroke mimic group and AIS after IV fluid	Kang, J. et al. [34]
Undetermined AIS: thrombotic (UND-AT) or embolic (UND-E)	SBV DBV	Before hydration therapy Up to 24 h testing	Scanning capillary viscometer (Hemovister) 1 s^−1^–300 s^−1^	Association of increased SBV and DBV with UD-AT	Oh, J. et al. [35]
AIS with >50% stenosis of the MCA and IST, AAE, LBO according to MRI DWI topography	HSV LSV	6 mL with EDTA within 24 h with EDTA Up to 24 h testing	Scanning capillary viscometer (Hemovister) 5 s^−1^–300 s^−1^	Blood viscosity was highest in patients with MCA-IST, followed by MCA-AAE and MCA-LBO. Patients with early neurological deteriorarion (END) had higher LSV and HSV. The association between END and LSV was higher in patients with MCA-LBO.	Woo, H.G. et al. [36]
AIS: CE, LAA, SVO, OD	SBV DBV	3 mL sample with EDTA prior to IV infusion Up to 24 h testing	Scanning capillary tube viscometer (BVD-PRO1) at 36 ± 0.5 °C 1 s^−1^–300 s^−1^	Increased DBV is associated with poor 3-mo functional outcome	Lee, M. et al. [37]
AIS: CE, LAA, SVO, UND	WBV, PV EA, ED	Sample with EDTA within 3 days after onset 4 h to 24 h testing	Brookfield DVIII viscometer at 37 °C 4.5 s^−1^–450 s^−1^ Laser ektacytometer (LORRCA)	No changes of BV and PV. Increased EA.	Uygun, G. et al. [38]
AIS: CE, LAA, SVO, UND	SBV DBV	3 mL sample with EDTA before hydration therapy	Scanning capillary viscometer (Hemovister) 5 s^−1^–300 s^−1^	Higher DBV in SVO and in old LI and microbleeds on MRI. Association of DBV with SBV, age, C-reactive protein and hypertension.	Noh, S.-M. [39]
AIS: wake-up stroke CE, LAA, SVO, OD, UD	BV Hct	Blood samples within 72 h from onset	Calculation of BV using Ht values	Higher BV was associated with wake-up stroke in elderly (>65 years) in the SVO group.	Okumura, M. et al. [40]

Abbreviations: AAE, artery-to-artery embolism; AIS, acute ischemic stroke; BV, blood viscosity; CE, cardioembolic; Cryp, cryptogenic; DBV, diastolic blood viscosity; EA, erythrocyte aggregation; ED, erythrocyte deformability; EDTA, ethylendiaminetetraacetic acid; Ht, hematocrit; HSV, high shear viscosity; IS, ischemic stroke; IST, in situ thrombo-occlusion; IV, intravenous; LAA, large-artery atherosclerosis; LBO, local branch occlusion; LI, lacunar infarction; LSV, low shear viscosity; MCA, middle cerebral artery; MRI DWI, diffusion-weighted magnetic resonance imaging; OD, other determined; SAO, small-artery occlusion; SBV, systolic blood viscosity; SI, silent infarction; SVO, small-vessel occlusion; TIAs, transient ischemic attacks; UND, undetermined; WBV, whole blood viscosity.

**Table 2:** Formula-based vs. instrument-measured blood viscosity.

Aspect	Formula-Based (e.g., De Simone)	Instrument-Based (e.g., Viscometer)
Basis	Estimated using hematocrit (Hct) and total protein (TP)	Directly measured from whole blood using physical devices
Common Formula	HSR: (0.12 × Hct) + 0.17 × (TP − 2.07) LSR: (1.89 × Hct) + 3.76 × (TP − 78.42)	Not applicable
Sample Requirements	Only standard lab values (Hct, TP)	2–6 mL of fresh whole blood; sometimes anticoagulated (EDTA)
Time to Result	Immediate (once lab values available)	3–30 min, depending on device and setup
Shear Rate Consideration	Static (208 s^−1^ or 0.5 s^−1^)	Dynamic; full shear profiles (e.g., 1–1000 s^−1^, oscillatory modes)
Sensitivity to Pathology	Limited to changes in Hct and TP	Sensitive to RBC deformability, aggregation, temperature, real-time changes
Cost/Equipment Needs	Very low; no additional equipment	Medium to high; requires specialized viscometers (e.g., SCTV, EMS, Brookfield)
Clinical Use Case	Rapid estimation when viscometers unavailable	Diagnostic confirmation, stroke mimic differentiation, therapy monitoring
Limitations	Cannot capture non-Newtonian properties of blood	May be limited by device accuracy, operator variability, and processing time

Hct—Hematocrit, TP—total proteins, HSR—high shear rate, LSR—low shear rate, EDTA—ethylenediaminetetraacetic acid, RB—red blood cell, SCTV—scanning capillary tube viscometer, EMS—electromagnetically spinning viscometer.

## Data Availability

No new data were created or analyzed in this study. Data sharing is not applicable to this article.
